# Full-Coverage Path Planning for Heterogeneous UUVs Using a Hybrid Detection Point Layout and a Dual-Chromosome Co-Evolutionary Genetic Algorithm

**DOI:** 10.3390/s26165195

**Published:** 2026-08-17

**Authors:** Fang Ji, Mengxi Shi, Weijia Feng, Xiang Ji, Xiao Xu

**Affiliations:** China Ship Research and Development Academy, Beijing 100101, China; shi.mx@outlook.com (M.S.);

**Keywords:** heterogeneous UUV path planning, full-coverage search, dual-chromosome co-evolutionary genetic algorithm, path length optimization, load balancing

## Abstract

To address the issue of unbalanced path allocation in multi-UUV cooperative operations under inhomogeneous ocean environments during full-coverage search missions, this paper proposes a heterogeneous UUV path planning method that integrates a hybrid waypoint deployment strategy with a dual-chromosome co-evolutionary genetic algorithm. First, heterogeneous UUVs are adaptively assigned to sub-regions according to the search value of the sea area, and a combination of Poisson sampling and Voronoi iterative refinement is adopted to complete the layout of detection points. Subsequently, connectivity-constrained K-means clustering is introduced to decompose the multi-traveling salesman problem (MTSP) into several independent TSP sub-problems. Finally, a dual-chromosome encoding scheme for task sequences and split points is designed, and a penalty matrix is incorporated into the fitness function to account for obstacle avoidance constraints, thereby establishing an integrated genetic-algorithm-based solution framework that incorporates both decomposition and obstacle avoidance. Simulation results demonstrate that the proposed method reduces the number of planned detection points by 12.4%, 12.8%, and 9.3% compared with baseline methods in circular, rectangular, and irregular sea areas, respectively, while the optimal path lengths are shortened by 5.8%, 4.5%, and 5.9%. Moreover, the cooperative mission time with four UUVs is reduced by 73.9%, 72.7%, and 71.2% relative to a single UUV, demonstrating an approximately linear speedup relative to the number of UUVs. Convergence analysis and extended experiments on 15 instances further confirm the algorithm’s solution stability and robustness under varying regional scales, shapes, and obstacle configurations. These results validate that the proposed approach not only reduces the number of deployment points and path cost, but also effectively balances obstacle avoidance and multi-robot load distribution.

## 1. Introduction

Ocean exploration and development are priorities for many nations, and unmanned underwater vehicles (UUVs) play a critical role in tasks such as marine survey, search and rescue, and environmental monitoring due to their high reliability, strong flexibility, and good robustness.

Full-coverage search path planning, a critical task for UUVs operating in unknown waters, aims to generate a collision-free, low-overlap, and low-energy-consumption traversal path that achieves complete coverage of the target area in complex environments [1]. In this task, a single UUV often encounters bottlenecks in exploration efficiency, robustness, and coverage range, whereas multi-UUV systems can significantly improve exploration performance through parallel cooperation [2]. By virtue of their advantages in timeliness, task parallelism, and fault tolerance, multi-UUV path planning methods have become a research hotspot for complex coverage tasks [3]. However, when scaling from a single UUV to multi-UUV cooperation for full-coverage missions, the complexity of path planning increases exponentially [4], and system performance is influenced by factors such as the area partitioning strategy [5], the initial deployment positions of the UUVs, and the coverage path planning algorithm [6].

Current mainstream full-coverage path planning approaches mainly include traditional geometric partitioning, intelligent optimization, and learning-based methods. Traditional geometric partitioning methods fully exploit regional symmetry and boundary smoothness, and often directly obtain theoretical lower bounds through geometric recursion, making them significantly more efficient than numerical search methods. Hungerford et al. [7] used Voronoi partitioning to divide the environment into several non-overlapping regions according to the initial positions of robots, achieving efficient solutions to the complete-coverage path planning problem. Vandermeulen et al. [8] proposed a partition-based heuristic method that reduces the number of turns in the coverage path by dividing the environment into long-strip rectangles whose width matches the robot’s coverage tool. Although traditional geometric partitioning is computationally efficient, its applicability is severely limited by the geometric regularity and physical homogeneity of the environment [9]. When the sea area is non-convex, contains obstacles such as reefs and islands, or exhibits differentiated search requirements across sub-regions, these methods often fail due to difficulties in satisfying physical constraints. In terms of intelligent optimization, Ni et al. [10] improved the K-means clustering algorithm to achieve more reasonable task area partitioning, but the partitioning quality degrades for non-convex regions, narrow terrains, and obstacle-rich environments, often fragmenting areas that should remain connected. Luo et al. [11] used fuzzy C-means clustering to decompose the coverage area, but did not consider obstacle barriers, which may cause a single cluster region to be disconnected. Gong et al. [12] introduced graph partitioning methods traditionally used in communications into multi-robot coverage path planning, reducing total path length and execution time, but the area partitioning lacks task balance constraints. Wu et al. [13] proposed a deep-reinforcement-learning-driven coverage path planning framework that uses probabilistic environment modeling and reward functions to enhance the real-time perception and decision-making capabilities of UAVs in complex marine environments. Li et al. [14] proposed a cooperative path planning algorithm based on multi-agent deep reinforcement learning, optimizing the completion time of multi-UAV dynamic target monitoring tasks. Ma et al. [15] proposed an improved non-dominated sorting genetic algorithm to optimize task allocation for multiple UAVs in complex environments. Yang et al. [16] established a task allocation algorithm for multiple UAVs based on a brain-inspired decision-making model, improving the efficiency of task allocation. Wang et al. [17] proposed a co-evolutionary hybrid-variable particle swarm optimization algorithm, which effectively solves the multi-UAV cooperative task allocation problem. However, learning-based methods often suffer from high computational complexity [18] and poor generalization ability [19].

In summary, existing methods suffer from the following common issues when addressing complex scenarios involving the coupling of inhomogeneous ocean environments, heterogeneous UUV clusters, and irregular obstacles: a disconnection between coverage strategy and resource allocation—the placement of detection points is not linked to the distribution of prior information, leading to insufficient coverage of high-value areas and waste of resources in low-value areas; task balancing and path optimization are separated, making it impossible to dynamically adjust the task load during path optimization and difficult to accommodate the detection capability differences of heterogeneous UUVs; and it is challenging to reconcile obstacle avoidance with dimensionality reduction—traditional geometric decomposition tends to produce fragmented sub-regions in obstacle-dense areas, while directly solving the MTSP using intelligent optimization algorithms faces a combinatorial explosion.

The core of the above issues lies in the fact that existing methods fail to incorporate task allocation and path planning into a unified framework for co-optimization. Notably, the genetic algorithm (GA), as a general-purpose tool for solving the MTSP, has been widely validated for its theoretical maturity and effectiveness. The MTSP is a generalization of the classical traveling salesman problem (TSP) and has been proven to be NP-hard [20,21], with extensive applications in robot path planning and logistics scheduling. GA is favored for its simplicity, ease of implementation, and strong global search capability [22], and numerous studies have confirmed its ability to produce high-quality approximate optimal solutions within acceptable time [23]. Among GA-based encoding strategies for the MTSP, dual-chromosome (or two-part-chromosome) encoding is considered one of the most effective schemes [24], in which one chromosome represents the visiting sequence of tasks and the other denotes the assignment of tasks to executors [25]. This encoding strategy has been widely adopted in cooperative task allocation and path planning problems for multi-UAV and multi-robot systems [22,25]. A systematic comparison of four mainstream MTSP encoding strategies further confirms that multi-chromosome encoding (including dual-chromosome encoding) significantly outperforms single-chromosome encoding in terms of solution quality [26]. Therefore, based on the dual-chromosome encoding genetic algorithm and targeting the specific requirements of complex marine scenarios, this paper proposes a heterogeneous UUV full-coverage path planning method. The main contributions of this paper are as follows:

(1) A hybrid detection point layout combining Poisson disk sampling and Voronoi diagram gap filling is proposed. It generates a spatially uniform initial point set and iteratively fills coverage gaps via Voronoi vertices, optimizing both the number of detection points and coverage rate without discretizing physical constraints.

(2) A cooperative co-evolutionary encoding strategy with “task sequence + split point” chromosomes is designed for heterogeneous UUV load balancing. The two chromosomes evolve independently within the same individual while sharing a unified fitness, enabling simultaneous optimization of path topology and task load distribution.

(3) An integrated GA-based framework combining decomposition and obstacle avoidance is constructed. Connectivity-constrained K-means clustering decomposes the obstacle-laden MTSP into independent TSP subproblems, while a penalty matrix embedded in the fitness function converts obstacle avoidance into continuous path costs, reducing solution complexity while ensuring collision-free paths.

Table 1 systematically compares our method with representative studies from five key dimensions: core solution algorithm, subproblem composition, obstacle avoidance capability, heterogeneous agent support, and environmental adaptability.

As shown in Table 1, most existing studies fail to simultaneously address obstacle avoidance, agent heterogeneity, and non-uniform task environments. In contrast, by integrating hybrid detection point layout, connectivity-constrained K-means decomposition, and dual-chromosome GA-based co-optimization, our method effectively balances path cost, heterogeneous agent constraints, and complex marine environmental factors.

## 2. Problem Formulation

The detection capability of a UUV is a function of the signal-to-noise ratio (SNR) received by its sonar system. According to the sonar equation [37], the SNR(P) received by the UUV at position P is given by:

(1) SNR(P)=SL−TL−(NL−DI)

where SL is the source level, TL is the transmission loss, NL is the ambient noise level, and DI is the directivity index.

The signal excess relevant to UUV detection requirements is written as:

(2) SE=SNR(P)−DT

where DT is the detection threshold. The value of SE directly determines the sonar’s probability of detecting a target.

It can be seen from Equations (1) and (2) that the sonar detection capability of UUVs varies with different locations, and this variation originates from the spatial changes in sonar performance and environmental parameters. Therefore, in full-coverage search tasks, not all areas are equally important. In fact, factors such as the prior distribution of possible target occurrence, the complexity of the marine environment, and mission priorities jointly determine the urgency and resource requirements for searching each sub-region. Hence, in this paper, we adopt the concept of search value to comprehensively characterize the importance of each position within the mission area. It should be noted that the search value can be quantified by various means, including the prior probability of target presence, environmental information entropy, or expert knowledge.

Based on the problem description above, the full-coverage search path planning problem can be formulated as the following mixed-integer programming model. Suppose m heterogeneous UUVs are required to perform a full-coverage search mission on n detection points, and the search value of each detection point is characterized by the value matrix V. Each UUV k(k=1,2,…,m) departs from a fixed starting point 0, traverses its assigned set of detection points, and terminates at its last task point (without returning to the start).

The decision variables are defined as follows:

$$x_{ijk}=\left\{\begin{array}{cc} 1, & \mathrm{if}\;\mathrm{UUV}\;k\;\mathrm{travels}\;\mathrm{from}\;\mathrm{detection}\;\mathrm{point}\;i\;\mathrm{to}\;\mathrm{detection}\;\mathrm{point}\;j,\\ 0, & \mathrm{otherwise}, \end{array}\right.\;i,j\in \{0,1,\ldots ,n\},\;i\neq j\;y_{ik}=\left\{\begin{array}{cc} 1, & \mathrm{if}\;\mathrm{detection}\;\mathrm{point}\;i\;\mathrm{is}\;\mathrm{assigned}\;\mathrm{to}\;\mathrm{UUV}\;k,\\ 0, & \mathrm{otherwise}, \end{array}\right.\;i\in \{1,\ldots ,n\},\;k\in \{1,\ldots ,m\}$$

Objective Function:

(3) $$\min\left(\max_{k\in \{1,\ldots ,m\}}\sum_{i=0}^{n}\sum_{j=0}^{n}\omega_{ij}\cdot x_{ijk}\right)$$

where $\omega_{ij}$ is the actual traversal cost from detection point i to detection point j, defined as:

(4) $$\omega_{ij}=\alpha d(i,j)$$

Here, d(i,j) is the Euclidean distance between points i and j, and α≥1 is the obstacle avoidance penalty factor. The min-max objective function is adopted to minimize the longest path among all UUVs, thereby achieving balanced task load distribution across heterogeneous UUVs and preventing any single UUV from becoming a performance bottleneck due to excessive workload.

The above optimization problem is subject to the following constraints:

Coverage constraint: Each detection point is visited by exactly one UUV.

(5) $$\sum_{k=1}^{m}y_{ik}=1,\;\forall i\in \{1,\ldots ,n\}$$

Flow conservation constraints: For each UUV, the number of arrivals equals the number of departures at each of its assigned detection points.

(6) $$\sum_{j=0}^{n}x_{ijk}=y_{ik},\;\forall i\in \{1,\ldots ,n\},\;\forall k$$

(7) $$\sum_{i=0}^{n}x_{ijk}=y_{jk},\;\forall j\in \{1,\ldots ,n\},\;\forall k$$

where the subscript 0 denotes the common starting point for all UUVs.

Subtour elimination constraints: To prevent disconnected closed subtours that are not connected to the starting point in any single UUV’s path, the Miller-Tucker-Zemlin (MTZ) formulation is adopted:

(8) $$u_{ik}-u_{jk}+n\cdot x_{ijk}\leq n-1,\;\forall i,j\in \{1,\ldots ,n\},\;i\neq j,\;\forall k$$

(9) $$1\leq u_{ik}\leq n,\;\forall i\in \{1,\ldots ,n\},\;\forall k$$

where $u_{ik}$ is an auxiliary variable denoting the position index of detection point i in the visiting sequence of UUV k.

Workload constraint: Each UUV is assigned at least one detection point to ensure that all UUVs participate in the mission.

(10) $$\sum_{i=1}^{n}y_{ik}\geq 1,\;\forall k\in \{1,\ldots ,m\}$$

Value-area adaptation constraint: Detection points in high-value areas are preferentially assigned to UUVs with smaller detection radii and higher detection precision (corresponding to effective detection radius $r_{H}$), while those in low-value areas are assigned to UUVs with larger detection radii suitable for wide-area coarse search (corresponding to effective detection radius $r_{L}$). It captures the matching relationship between heterogeneous UUV configurations and regional search values, representing a key feature that distinguishes our method from existing studies that assume homogeneous UUVs.

The above mathematical model and constraint set constitute a rigorous formalization of the full-coverage search path planning problem. This formalization provides a clear mathematical foundation for the connectivity-constrained clustering decomposition strategy, the dual-chromosome encoding, and the obstacle avoidance penalty mechanism introduced in Section 3.2.

## 3. Model Construction

The proposed method in this paper aims to achieve full coverage of the search area and minimization of path cost for coverage search tasks with uneven distribution of search value in complex marine environments. The overall framework consists of two core modules: a Poisson-Voronoi hybrid detection point layout, and a genetic path planning based on connected-domain decomposition and dual-chromosome co-evolution.

### 3.1. Poisson-Voronoi Hybrid Detection Point Layout

In this section, to address the problems of improper density distribution of randomly placed sampling points and the high cost of filling sampling gaps in inhomogeneous ocean environments, a Poisson-Voronoi hybrid sampling strategy is proposed.

#### 3.1.1. Adaptive Configuration of Heterogeneous Unmanned Underwater Vehicles

To achieve a balance between search efficiency and coverage accuracy, this paper proposes an adaptive heterogeneous configuration method based on the distribution characteristics of non-uniform marine environments. This method abandons the traditional uniform deployment strategy of UUVs, divides the search area into high-value and low-value sub-regions according to environmental complexity, and accordingly allocates heterogeneous UUV resources with different search radii $\left(r_{H},r_{L}\right)$ in a dynamic manner.

Using the search value matrix $P\in {\left[0,1\right]}^{m\times n}$, where the element $p_{\mathrm{ij}}$ denotes the search value at coordinate (i,j), we first divide the entire area into $N_{X}\times N_{Y}$ non-overlapping sub-region grids along the X and Y axes. The global mean $\mu_{global}$ is calculated as the partition baseline:

(11) $$\mu_{global}=\frac{1}{M\times N}\sum_{i=1}^{M}\sum_{j=1}^{N}p_{\mathrm{ij}}$$

For the k-th sub-region (k=1,2,…,K), extract the matrix $P_{k}$ and calculate its local mean $\mu_{k}$:

(12) $$\mu_{k}=\frac{1}{\left|P_{k}\right|}\sum_{p\in P_{k}}p$$

Based on the relative magnitude between the local mean $\mu_{k}$ and the global mean $\mu_{global}$, a binary classification criterion is established to divide the sub-regions into “high-value areas” and “low-value areas”. This criterion reflects the significance of each sub-region relative to the global environment. In low-value areas, where the mean is below the global baseline ($\mu_{k}<\mu_{global}$), the environmental features are relatively simple or the likelihood of target occurrence is uniform across the region; in high-value areas, where the overall level is higher ($\mu_{k}>\mu_{global}$), it generally implies that high precision (small-radius) UUVs should be allocated for focused search and inspection. For the rare boundary case (typically occurring only at numerical rounding errors) where $\mu_{k}=\mu_{global}$—which typically occurs only at numerical rounding errors—we classify the sub-region as a high-value area by default to ensure coverage redundancy for potentially critical targets.

This mechanism ensures that UUVs with smaller search radii are assigned to areas with dense high-value targets for refined search, while UUVs with larger search radii are deployed in low-value areas for coarse-grained coverage, thereby optimizing the number of detection points while maintaining coverage.

#### 3.1.2. Poisson Disk Sampling

The algorithm independently performs Poisson disk sampling within each category of sub-region to generate an initial point set $s_{0}$. Poisson disk sampling introduces a geometric constraint on top of random sampling—the Euclidean distance between any two points must be greater than a given minimum radius $r_{\min}>0$, i.e.,

(13) $${∥s_{i}-s_{j}∥}_{2}\geq r_{min},\forall i\neq j$$

The core of Poisson disk sampling lies in the setting of the minimum radius $r_{\min}$. The algorithm adopts a value $r_{\min}=r_{L}$ or $r_{\min}=r_{H}$ that is determined by the assigned UUV search radius: in high-value sub-areas, where smaller-radius UUVs are allocated, a smaller Poisson sampling radius is used to generate denser detection points; the opposite holds for low-value sub-areas. This achieves a positive correlation between detection point density and search value.

Compared with random sampling, Poisson disk sampling offers the following advantages: (1) High spatial utilization—the minimum distance constraint pushes points apart in space, forming a local structure close to hexagonal close packing, which greatly reduces voids and overlap. (2) Blue noise characteristics—strong high-frequency components and weak low-frequency components, meaning that no low-frequency patches or streaks appear in the sampling result. (3) Controllable point density—the sampling density can be directly controlled by adjusting the parameter $r_{min}$, enabling approximate control over the number of points.

#### 3.1.3. Voronoi Iterative Filling

Poisson disk sampling inevitably leaves some tiny voids in the space where no new sampling point can be accommodated. To achieve full coverage of the mission area, the algorithm introduces an iterative filling mechanism by constructing the Voronoi diagram of the point set [38]. Define the Voronoi cell as $V_{i}=\left\{x\in R^{2}\sim |x-s_{i}\leq \;∥x-s_{j}∥,\forall j\neq i\right\}$, the iterative procedure is as follows:

(1) Extract all Voronoi vertices V  and calculate the maximum gap according to the following formula:

(14) $$d_{\max}=\max_{v\in V}\min_{s\in S}∥v-s∥$$

(2) If $d_{\max}>r_{L}$ (or $d_{\max}>r_{H}$), add the corresponding vertex v to the detection point set $s_{0}$;

(3) Repeat steps (1) and (2) until $d_{\max}\leq r_{L}$ (or $d_{\max}\leq r_{H}$). This mechanism ensures precise supplementation in the tiny voids left by Poisson sampling, avoiding blind densification.

**Figure 1:**
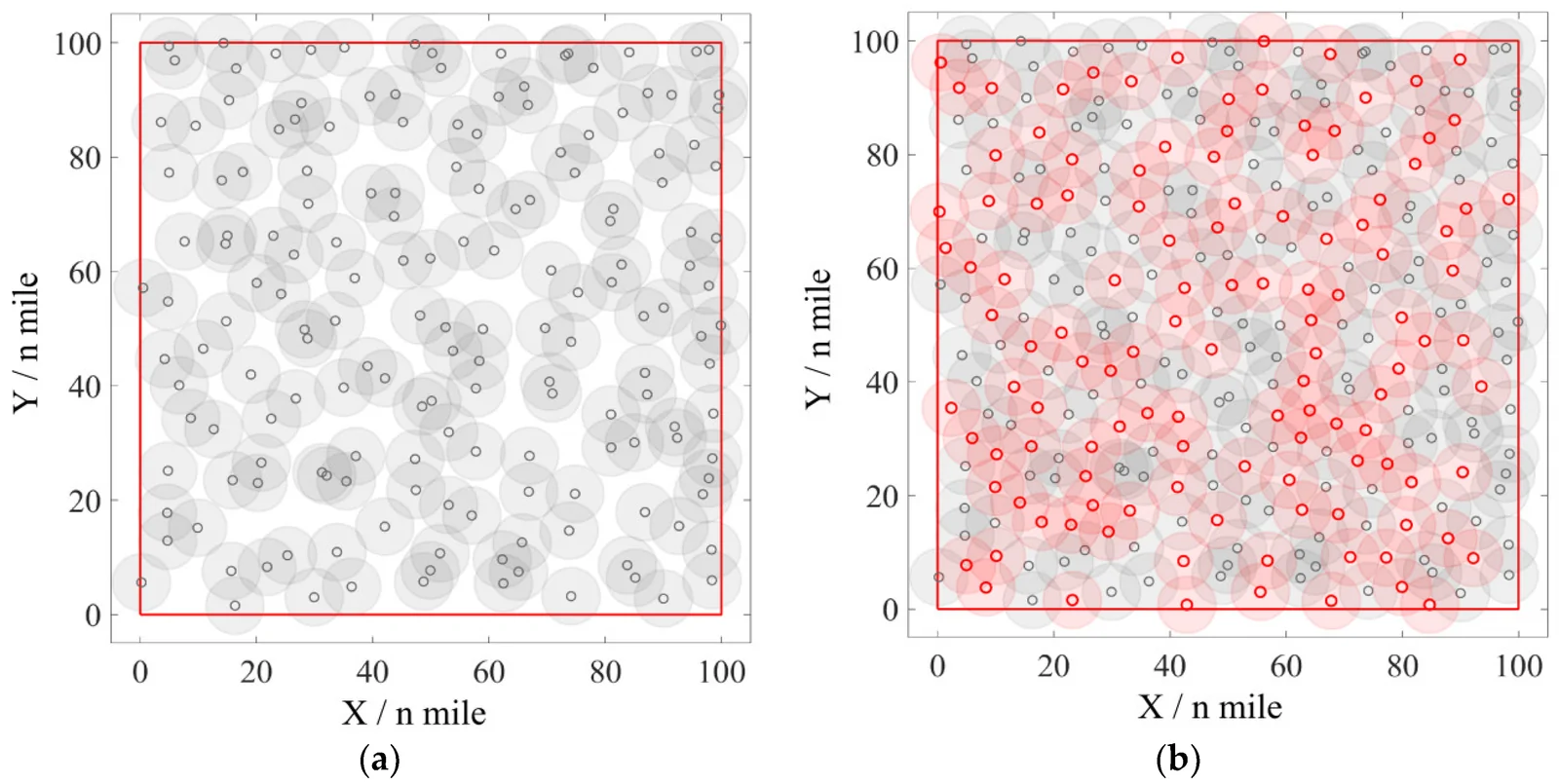
Comparison of coverage (**a**) before and (**b**) after Voronoi filling.

In a sea area of 100 n mile by 100 n mile, Poisson disk sampling was performed with a search radius of 5 n mile, yielding 169 detection points and achieving a coverage rate of 83% for the target mission area. The sampling result is shown in Figure 1a. By ensuring that the distance between any two sampling points is no less than a preset minimum radius, this sampling method achieves a uniform spatial distribution of the point set and effectively avoids point clustering. However, as can be seen from Figure 1a, although Poisson disk sampling largely attains coverage uniformity over the target mission area, gaps still exist locally. To address this issue, this paper introduces an iterative filling strategy based on the Voronoi diagram. Figure 1b illustrates the effect of this filling process—the Voronoi filling mechanism adds 120 new detection points (in red), which are primarily distributed in the gaps between the original Poisson points (in gray), achieving full coverage of the target mission area while controlling search range overlap.

It should be noted that, although the Voronoi diagram is used for gap detection and filling during detection point deployment, it serves merely as a gap-filling tool rather than for spatial partitioning. This contrasts with classical Voronoi partition-based methods, which rely on the assumption of uniform detection conditions across sub-regions and thus cannot account for heterogeneous search values or adapt to UUVs with different detection capabilities. Therefore, classical Voronoi methods are not directly applicable to our scenario of inhomogeneous marine environments with heterogeneous UUVs. Our method instead builds upon Voronoi geometry, combining it with Poisson sampling and value-guided strategies to develop an improved approach tailored for heterogeneous and non-uniform scenarios.

### 3.2. Dual-Chromosome Co-Evolutionary Genetic Algorithm

After completing the deployment of detection points, it is necessary to plan the optimal access sequence of the detection points for the UUV swarm. This problem belongs to a Min-Max type Multiple Traveling Salesman Problem (MTSP) variant [39] with the optimization objective of minimizing the longest path length. Each UUV departs from a fixed starting point, traverses its assigned points, and terminates at its last task point. To reduce the computational complexity of solving the MTSP and achieve task load balancing, this paper introduces a connected-component decomposition strategy before applying the genetic algorithm: for the set of points to be visited by each type of UUV, K-means clustering is used to partition it into a number of spatially connected sub-regions equal to the number of UUVs, and a connectivity check is performed on each cluster. If the detection points within a cluster are divided into multiple disconnected components due to obstacle obstruction, these components are reassigned to other clusters that are spatially closest and reachable. This strategy decouples the original MTSP into multiple independent Traveling Salesman Problems (TSP), greatly reducing the search dimension of the genetic algorithm and ensuring the spatial aggregation of task points for each UUV. On this basis, this paper improves the genetic algorithm in terms of chromosome encoding mechanism and fitness function. The specific designs are as follows:

#### 3.2.1. Dual-Chromosome Encoding Mechanism and Hybrid Genetic Operator Design

The task sequence chromosome is a permutation of the indices of the detection points to be visited, 

$\sigma =(\sigma_{1},\sigma_{2},\ldots ,\sigma_{n})$, where n is the number of detection points excluding the starting point, and it determines the visit order of the detection points. The split point chromosome is a set of m−1 strictly increasing positive integers, $s=\{s_{1}<s_{2}<\ldots <s_{m-1}\}$, with $1\leq s_{i}\leq n$, which is used to partition the sequence σ into m segments and assign them to the respective UUVs. The task sequence of the i-th UUV consists of the elements from $\sigma_{s_{i-1}+1}$ to $\sigma_{s_{i}}$ (defining $s_{0}=0,s_{m}=n$).

To address the solution feasibility constraints (i.e., no duplicate detection points across UUVs) and the multi-objective balancing problem (i.e., balancing the number of detection points per UUV) faced by traditional genetic algorithms, this paper designs a neighborhood-constrained crossover operator for the task sequence chromosome and a split point perturbation mutation operator for the split point chromosome. The two operators are respectively responsible for exploring the global topological structure and fine-tuning the local task load.

The specific operation of the neighborhood-constrained crossover operator is as follows: let the parent individuals be $P_{1}=\{p_{11},p_{12},\ldots ,p_{1n}\}$ and $P_{2}=\{p_{21},p_{22},\ldots ,p_{2n}\}$. Randomly generate two crossover points $x_{1},x_{2}\in [0,n]$ with $x_{1}<x_{2}$. Extract the segment $S=P_{1}[x_{1}:x_{2}]$ and directly map it to the corresponding positions in the offspring, marking these positions as occupied. Traverse parent $P_{2}$, and sequentially fill the vacant positions in the offspring with the detection points that do not appear in segment $S=P_{1}[x_{1}:x_{2}]$, in the order they occur in parent $P_{2}$. This operator effectively inherits the optimization results of the parent in a local region by forcibly preserving a continuous path segment from the parent, thus avoiding the destruction of the path structure. At the same time, it utilizes the order information of the other parent to complete the sequence, ensuring no duplication or omission of detection points.

The specific operation of the split point perturbation mutation operator is as follows: for a split point $s_{i}$ (the task boundary between the i-th and (i+1)-th UUV), the mutation direction is determined according to the load difference between the two adjacent UUVs:

(15) $$\delta_{i}=\left\{\begin{array}{c} +1\\ -1\\ rand(\{-1,+1\}) \end{array}\begin{array}{c} L_{i}>L_{i+1}\\ L_{i}<L_{i+1}\\ L_{i}=L_{i+1} \end{array}\right.$$

The mutated split point is ${s_{i}}^{\prime }=s_{i}+\delta_{i}$. The function of this operator is to balance the path lengths of the UUVs by fine-tuning the split point positions to alter the task allocation boundaries, thereby achieving local fine-grained search and dynamic load balancing.

During the genetic optimization stage, the neighborhood-constrained crossover operator acts on the task sequence chromosome, while the split point perturbation mutation operator acts on the split point chromosome. The two chromosomes form a co-evolutionary relationship within the same evolving individual: the task sequence chromosome defines the global topological order of the detection points, providing the “segmentation material” for the split point chromosome; the split point chromosome, in turn, dynamically adjusts the task boundaries through perturbation mutation based on the feedback of each UUV’s path length derived from the current sequence. The two are coupled through a shared fitness function (see below): when the sequence chromosome explores a new path order, the split point chromosome responds immediately to balance the load; conversely, adjustments to the split points also change the evaluation result of the sequence chromosome, guiding it to evolve toward a more balanced topology. This co-evolutionary mechanism differs from the traditional two-stage approach of fixing the assignment first and then optimizing the order in MTSP solving, and achieves simultaneous optimization of task allocation and path planning.

**Figure 2:**
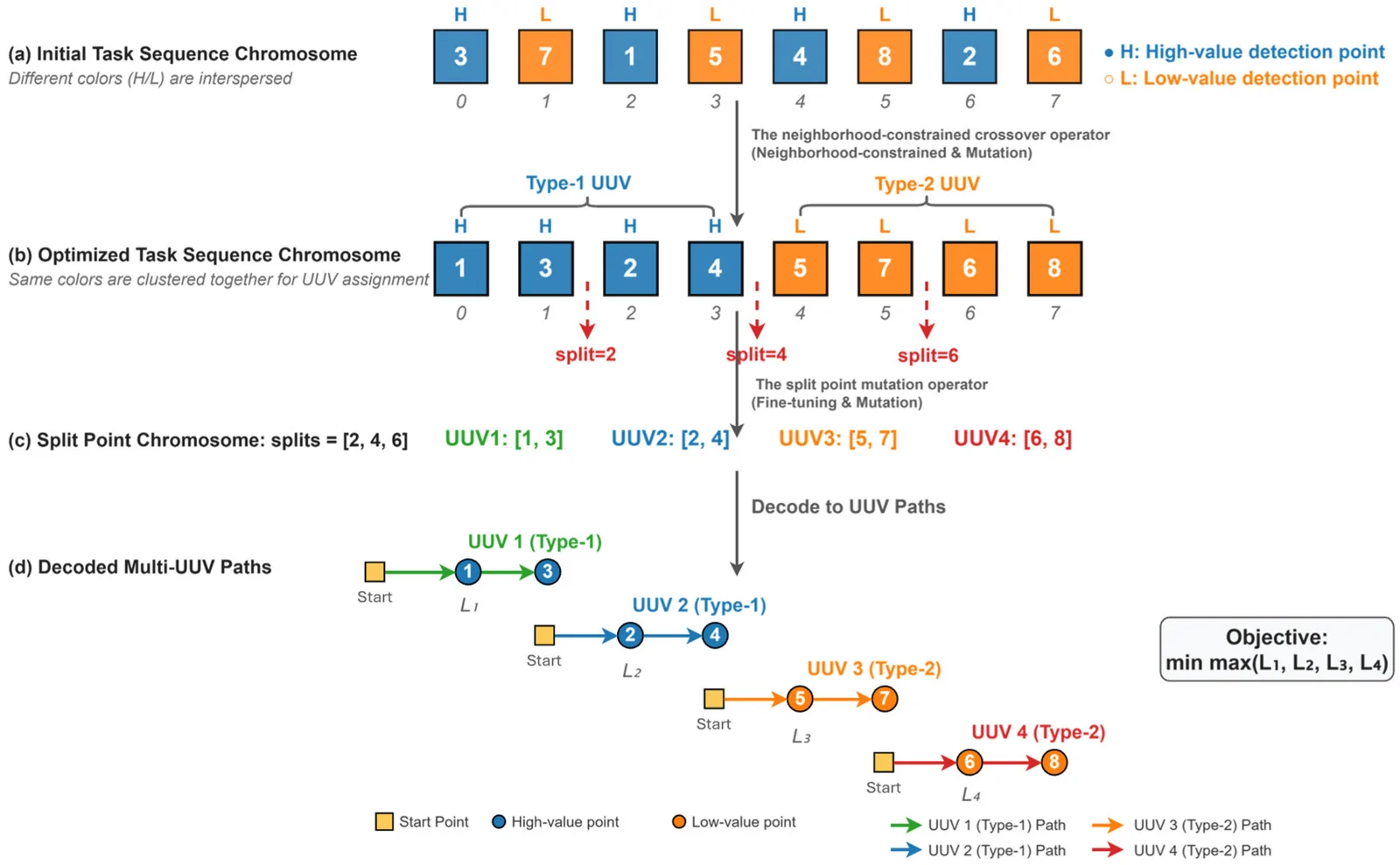
Illustration of the dual-chromosome decoding process.

To illustrate the dual-chromosome decoding mechanism, Figure 2 presents a step-by-step example. The four levels sequentially show: (a) the initial interleaved detection point arrangement; (b) the clustered task sequence chromosome after the neighborhood-constrained crossover operator; (c) the balanced split point chromosome after the split point mutation operator; and (d) the final decoded paths for each UUV.

#### 3.2.2. Construction of Fitness Function Based on Penalty Matrix

Considering the issue of efficiency redundancy of unmanned underwater vehicles (UUVs) in already-searched mission areas and the problem of passage obstruction caused by local obstacles, this paper introduces a penalty matrix into the fitness function to modify the path cost in the form of a soft constraint. The penalty matrix $\omega_{uv}$ is defined as follows:

(16) $$\omega_{uv}=\left\{\begin{array}{cc} \alpha \cdot d_{uv},if\;\bar{P_{u}P_{v}}\cap \Omega_{overed}\neq \emptyset & \left(\alpha >1\right)\\ \alpha \cdot d_{uv},if\;\bar{P_{u}P_{v}}\cap \Omega_{overed}=\emptyset & \left(\alpha =1\right) \end{array}\right.$$

where α is the penalty factor that controls the strength of obstacle avoidance. At α=1, the path cost reduces to pure Euclidean distance, and the algorithm minimizes only the travel distance. At α>1, an extra cost is added to paths that intersect or approach obstacles, encouraging the selection of collision-free routes with sufficient clearance. Here, $d_{uv}=\mathrm{dist}(P_{u},P_{v})$ denotes the Euclidean distance between detection points $P_{u}$ and $P_{v}$. By converting the binary obstacle constraint (passable/impassable) into the penalty factor α, the penalty matrix $\omega_{uv}$ effectively represents the actual cost of traveling from detection point $P_{u}$ to $P_{v}$. This allows the genetic algorithm to naturally develop a preference for obstacle-avoiding paths through cost comparison during the evolutionary process, without the need for complex feasibility verification at the decoding stage.

Let $n_{i}$ be the number of detection points assigned to the i-th UUV. The total path cost is defined as the sum of the modified distances:

(17) $$C\left(\pi^{\left(i\right)}\right)=\overset{n_{i}-1}{\underset{j=0}{\sum }}\omega_{\pi_{j}^{\left(i\right)},\pi_{j+1}^{\left(i\right)}}$$

where $\pi^{\left(i\right)}$ denotes the permutation (visiting order) of the detection points assigned to the i-th UUV. For a given permutation $\pi^{\left(i\right)}$, the path cost $C\left(\pi^{\left(i\right)}\right)$ is the cumulative sum of traversal costs incurred by visiting the detection points in that specified order. The core optimization objectives of the genetic algorithm is to iteratively search for and evolve $\pi^{\left(i\right)}$ to minimize the maximum cost among all paths (Min-Max). Therefore, the fitness value of an individual is defined as:

(18) $$f\left(\mathrm{ind}\right)=\frac{1}{\overset{m}{\underset{i=1}{\max}}C\left(\pi^{\left(i\right)}\right)+\varepsilon }$$

where $\varepsilon =10^{-8}$ is a tolerance error to prevent division by zero.

This fitness function serves to convert environmental constraints and path constraints into numerical costs. Specifically, by assigning high penalty weights to obstacles and already-searched mission areas, the penalty matrix first guides the genetic algorithm to automatically avoid these regions during the search process. Then, combined with the Min-Max optimization objective, the path length differences among UUVs are mapped into an additional load penalty term to suppress excessively long single paths. Thus, during the fitness evaluation stage, obstacle avoidance safety, detection deduplication, and load balancing for the heterogeneous swarm are achieved simultaneously.

## 4. Experiments and Analysis

In this study, all simulations are implemented in Python 3.9. The two-stage random search planning model proposed by Wang et al. [40] is selected as the baseline method. Specifically, the baseline method proposed by Wang et al. [40] consists of two stages. In the first stage, random sampling is performed within the target sea area to generate an initial set of detection points, and their positions and density are iteratively refined to achieve effective coverage of the mission region. In the second stage, based on the generated point set, a standard genetic algorithm is employed to solve the single-UUV TSP, yielding the shortest route that traverses all detection points. The overall architecture of this baseline can be summarized as a two-stage paradigm of “random sampling + genetic algorithm.”

The selection of this model is mainly based on the following consideration: the two-stage framework of “sampling + path planning” of this model is consistent with the basic architecture of the proposed method. This ensures that the comparative experiment can exclude interference from differences in algorithm architecture, focus on verifying the effectiveness of the hybrid detection point layout method and the dual-chromosome encoding mechanism proposed in this paper, and quantify the specific contributions of the improvement measures in reducing detection redundancy and optimizing path cost. To validate the applicability of the algorithm in optimal scenarios for traditional geometric partitioning methods, this paper selects rectangular and circular sea areas as typical simulation scenarios.

To ensure the reproducibility and fairness of the experiments, all key parameters of the proposed method are systematically listed in Table 2. These values were determined through preliminary sensitivity analysis and kept consistent across all experimental scenarios (circular, rectangular, and irregular sea areas). For the baseline method [40], the parameter settings follow the original configuration reported in the literature, with the UUV search radius set to 5 n mile to align with the comparative conditions.

### 4.1. Circular Sea Area

**Figure 3:**
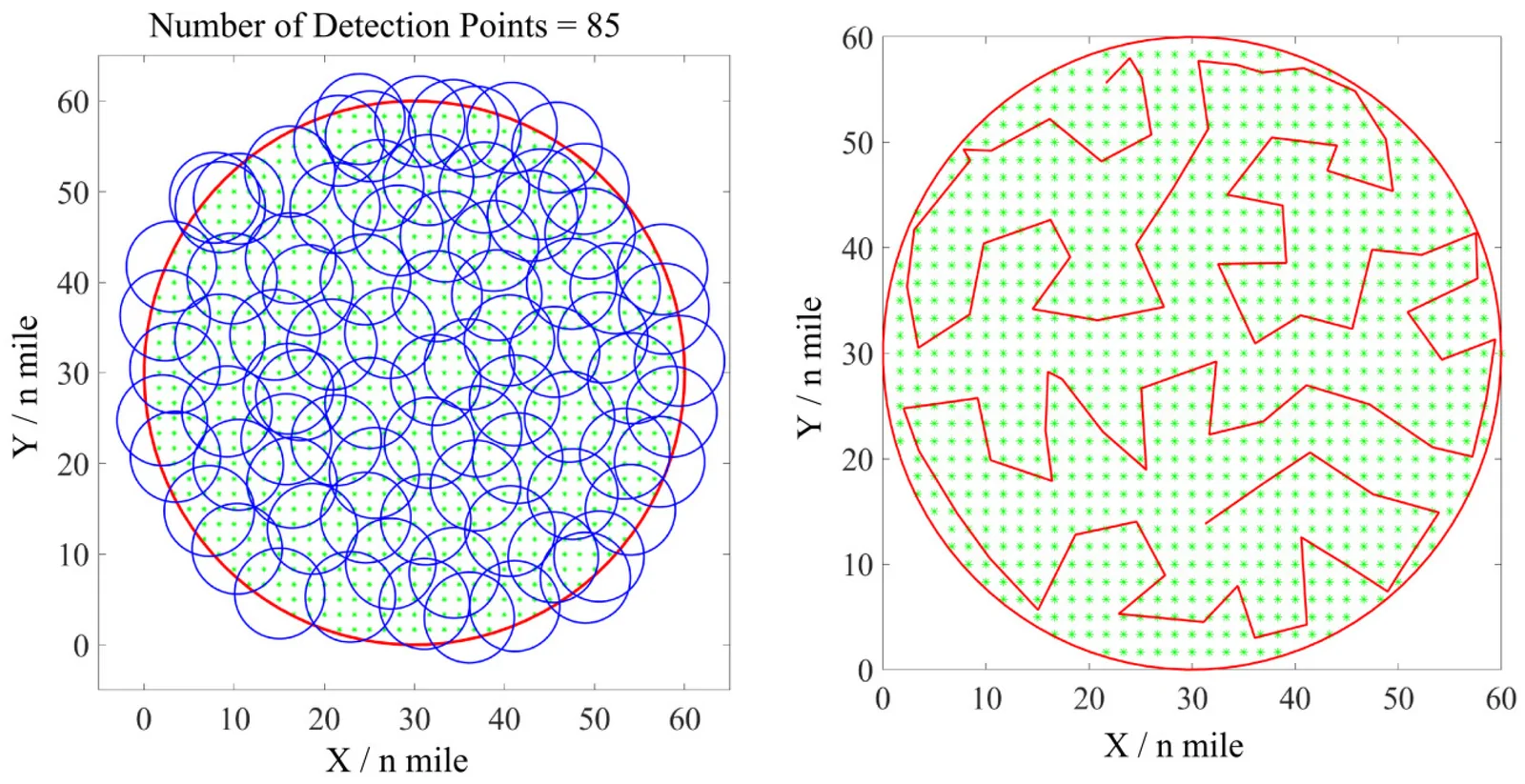
Coverage search scheme and search path in the circular sea area.

The radius of the circular simulation sea area is set to 30 n mile. In this task scenario, the baseline method plans a full-coverage search scheme with 97 detection points and an optimal search path length of 456.0 n mile. The proposed method achieves a full-coverage search scheme with 85 detection points and an optimal search path length of 429.5 n mile. The coverage search scheme and search path are shown in Figure 3.

Table 3 presents a comparison of the baseline method and the proposed method in completing the full-coverage search task in the circular sea area. It can be seen that, compared with the baseline method, the proposed method reduces the number of detection points by 12.4% and the optimal path length by 5.8%.

**Figure 4:**
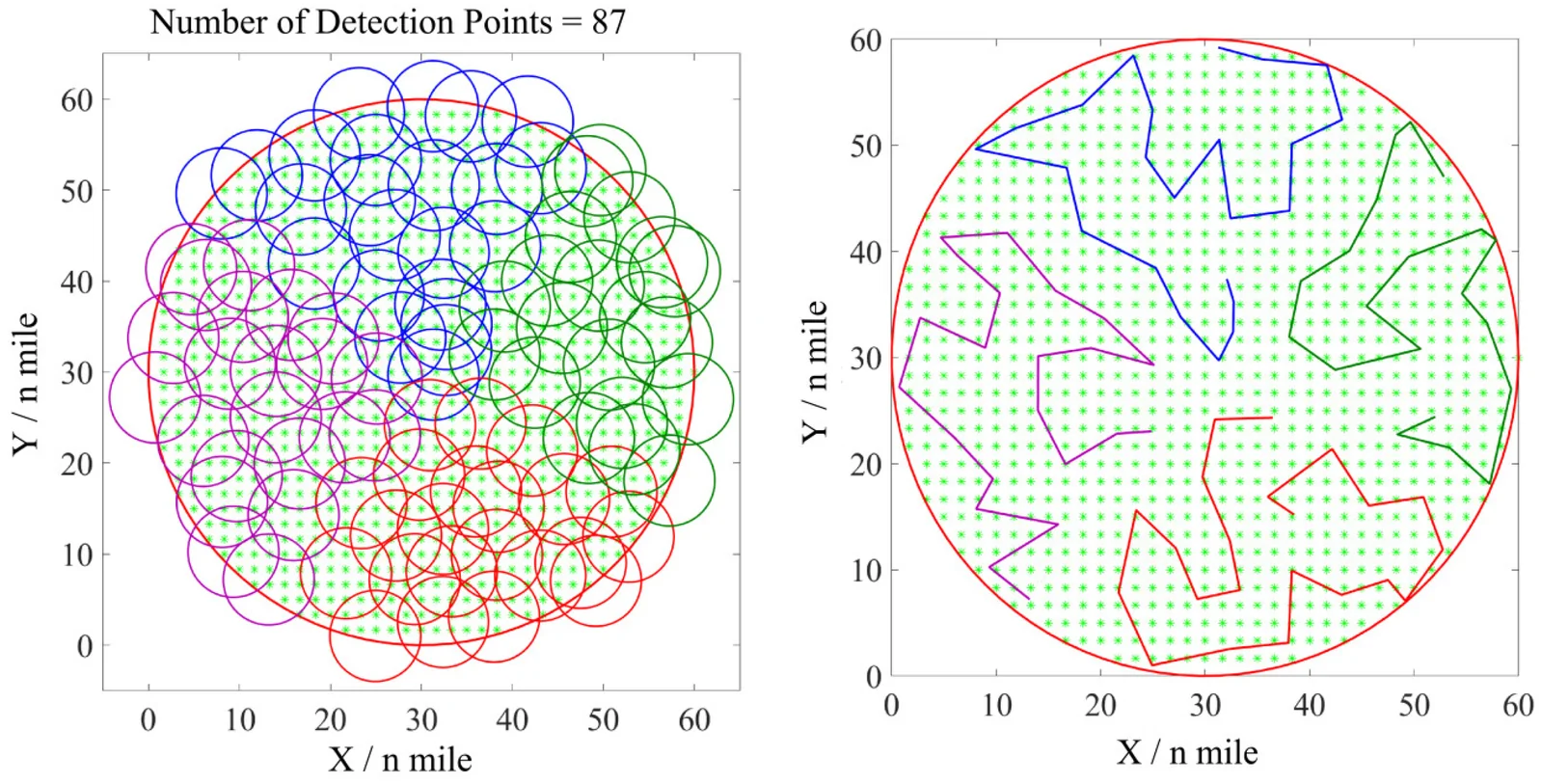
Multi-UUV coverage search scheme and search paths in the circular sea area.

Compared with a single UUV, the task parallelism of multi-UUV coverage search path planning enables better time efficiency. In the aforementioned circular sea area, four heterogeneous UUVs (two of each type) are used for cooperative search. The number of planned detection points is 87 (slightly different from 85 in the single-UUV scenario, due to the randomness of Poisson sampling; the median of multiple runs is adopted in this paper). The path lengths of the UUVs are 111.9 n mile, 108.7 n mile, 96.1 n mile, and 109.7 n mile, respectively, with a total path length of 426.4 n mile. The coverage search scheme and the search paths are shown in Figure 4. Compared with the full-coverage task scheme of the baseline method, the total number of detection points is reduced by 10.3%, and the total path length is reduced by 6.5%. When the speed of UUVs is fixed, the cooperative mission time is determined by the longest path among the UUVs. Compared with the single-UUV coverage search scheme, the four-UUV cooperative operation reduces the completion time for full coverage by 73.9%.

**Figure 5:**
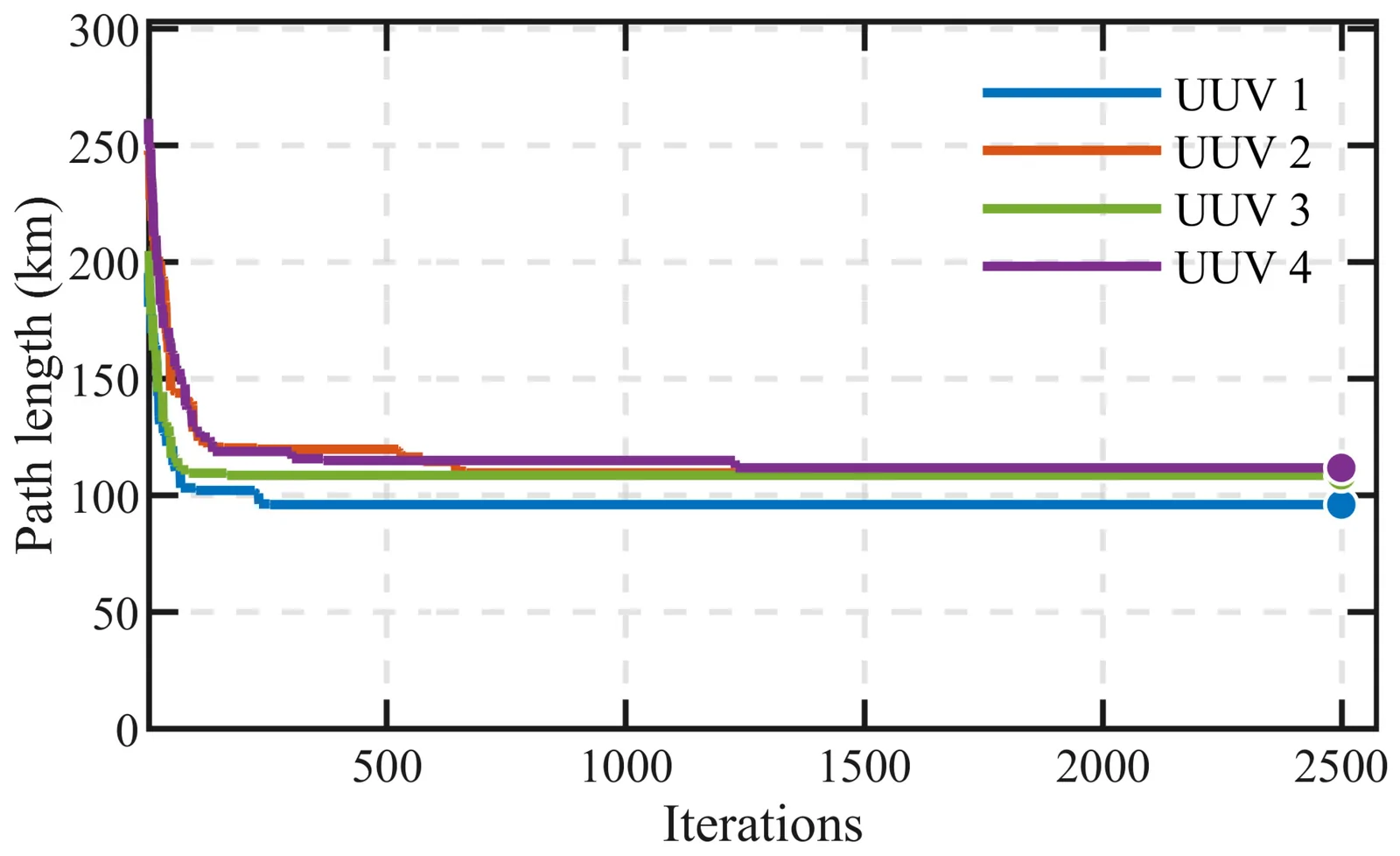
Convergence curve of the genetic algorithm in the circular sea area.

Furthermore, to validate the convergence performance of the algorithm, Figure 5 presents the variation curve of the fitness value (i.e., the maximum path length) during the genetic algorithm iterations in the circular sea area (R = 30 n mile). As clearly shown in the figure, within approximately 700 generations, the fitness value drops rapidly from about 250 n mile to approximately 120 n mile; after 700 generations, the curve gradually stabilizes and fully converges to about 110 n mile by around 1250 generations. This convergence behavior demonstrates that the genetic algorithm exhibits favorable search efficiency and solution stability in circular sea area scenarios, and is capable of obtaining stable near-optimal solutions at an acceptable computational cost.

Additionally, the path lengths of the four UUVs in the converged solution are 111.9 n mile, 108.7 n mile, 96.1 n mile, and 109.7 n mile, yielding a standard deviation of 6.4 n mile, indicating that the algorithm achieves well-balanced task allocation among heterogeneous UUVs in the circular scenario.

### 4.2. Rectangular Sea Area

**Figure 6:**
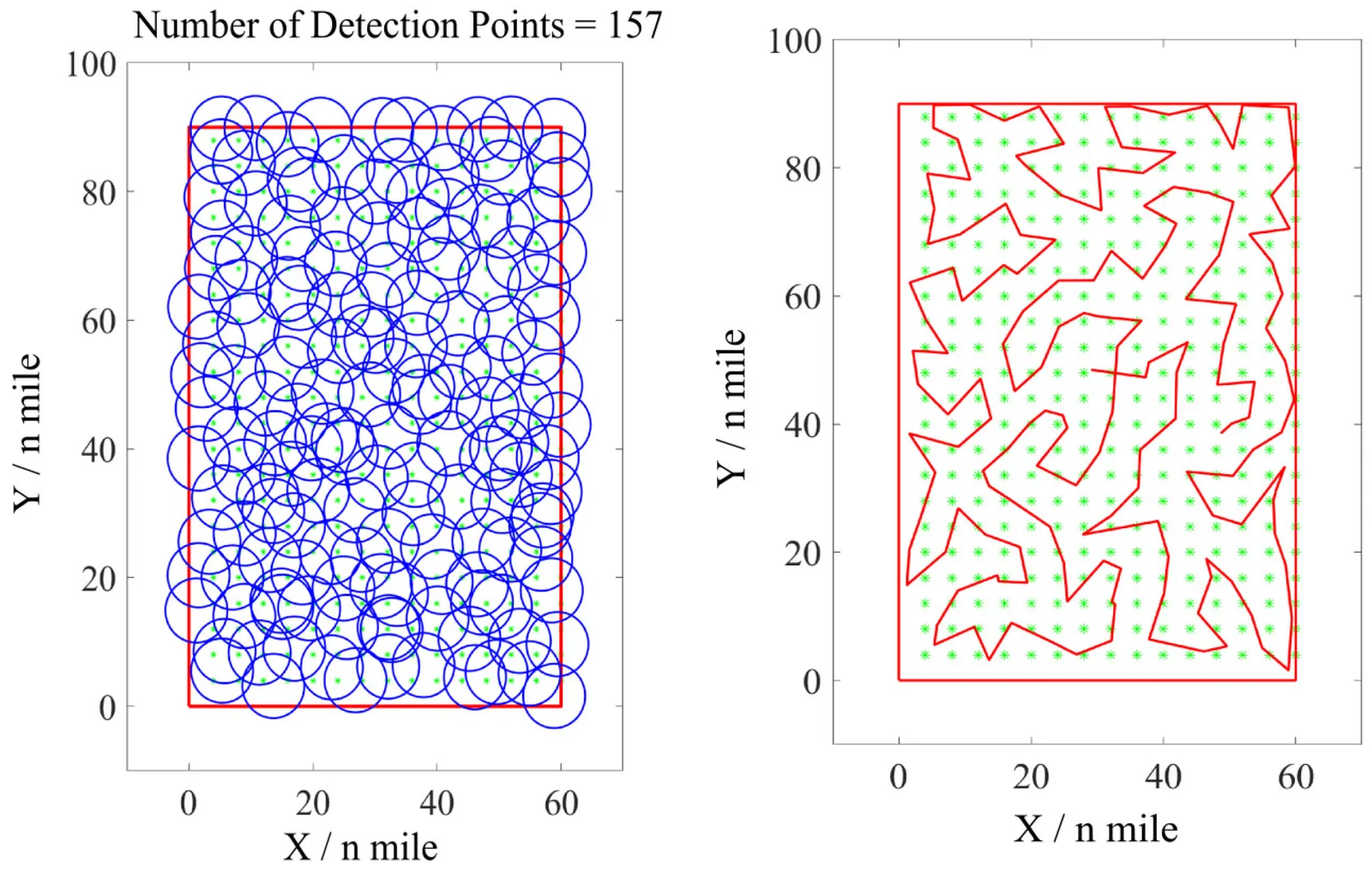
Coverage search scheme and search path in the rectangular sea area.

The length and width of the rectangular sea area are set to 90 n mile and 60 n mile, respectively. The baseline method plans a coverage search scheme with 180 detection points in this scenario, yielding a shortest path length of 883 n mile. The proposed method computes a full-coverage search scheme with 157 detection points and plans a shortest path of 843 n mile. The coverage search scheme and search path are shown in Figure 6.

Table 4 presents a comparison of the baseline method and the proposed method in completing the full-coverage search task. It can be seen that, compared with the baseline method, the proposed method reduces the number of detection points by 12.8% and the optimal path length by 4.5%.

**Figure 7:**
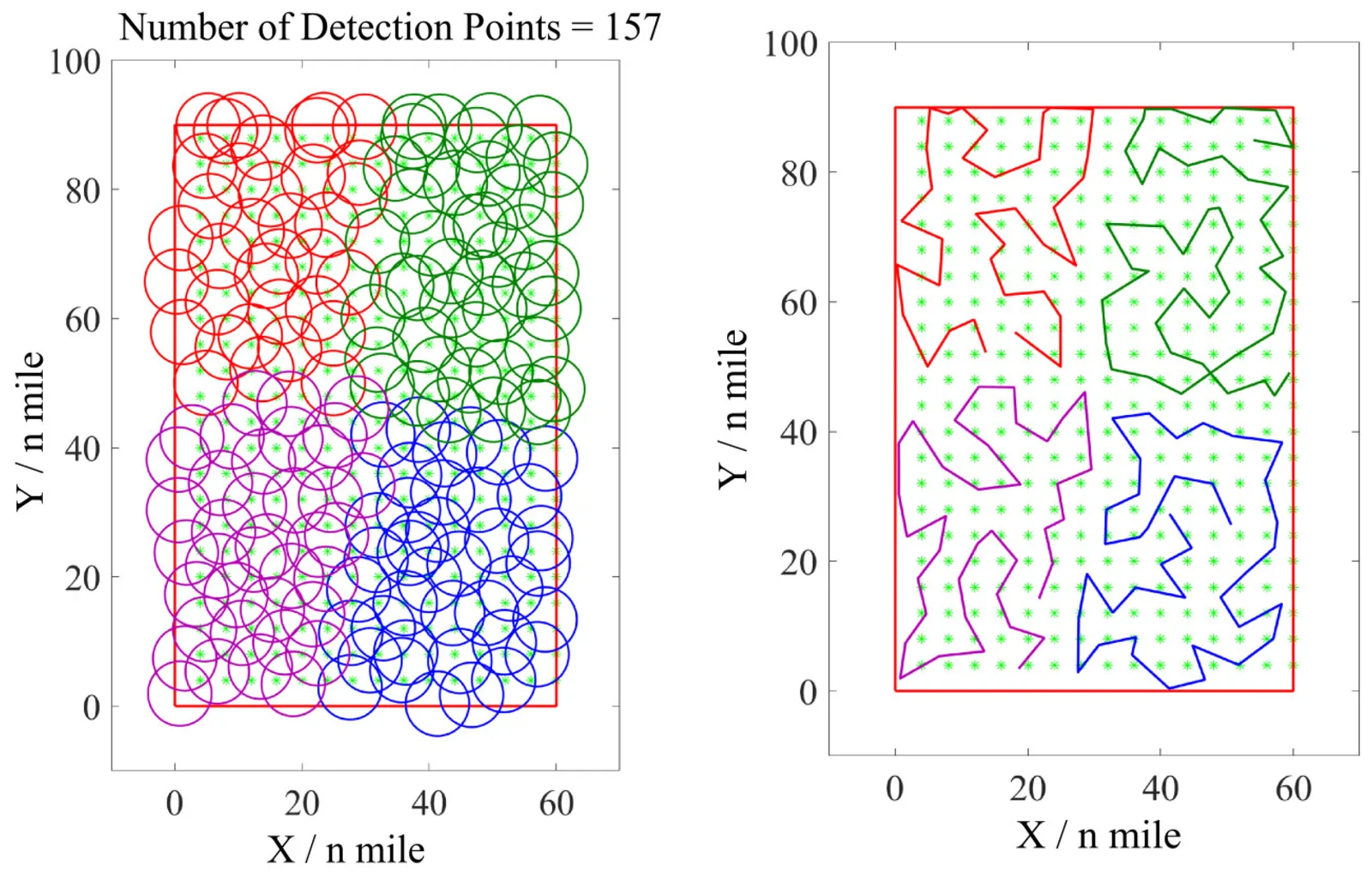
Multi-UUV coverage search scheme and search paths in the rectangular sea area.

In the aforementioned rectangular sea area, four heterogeneous UUVs (two of each type) are used for cooperative search. The proposed method plans 157 detection points. The path lengths of the individual UUVs are 211.9 n mile, 189.6 n mile, 230.3 n mile, and 206.1 n mile, respectively, with a total path length of 837.9 n mile. The coverage search scheme and the search paths are shown in Figure 7. Compared with the full-coverage task scheme of the baseline method, the proposed method reduces the total number of detection points by 12.8% and the total path length by 5.1%. When the UUVs travel at a fixed speed, the cooperative operation with four UUVs reduces the completion time for full coverage by 72.7% compared with the single-UUV coverage search scheme.

**Figure 8:**
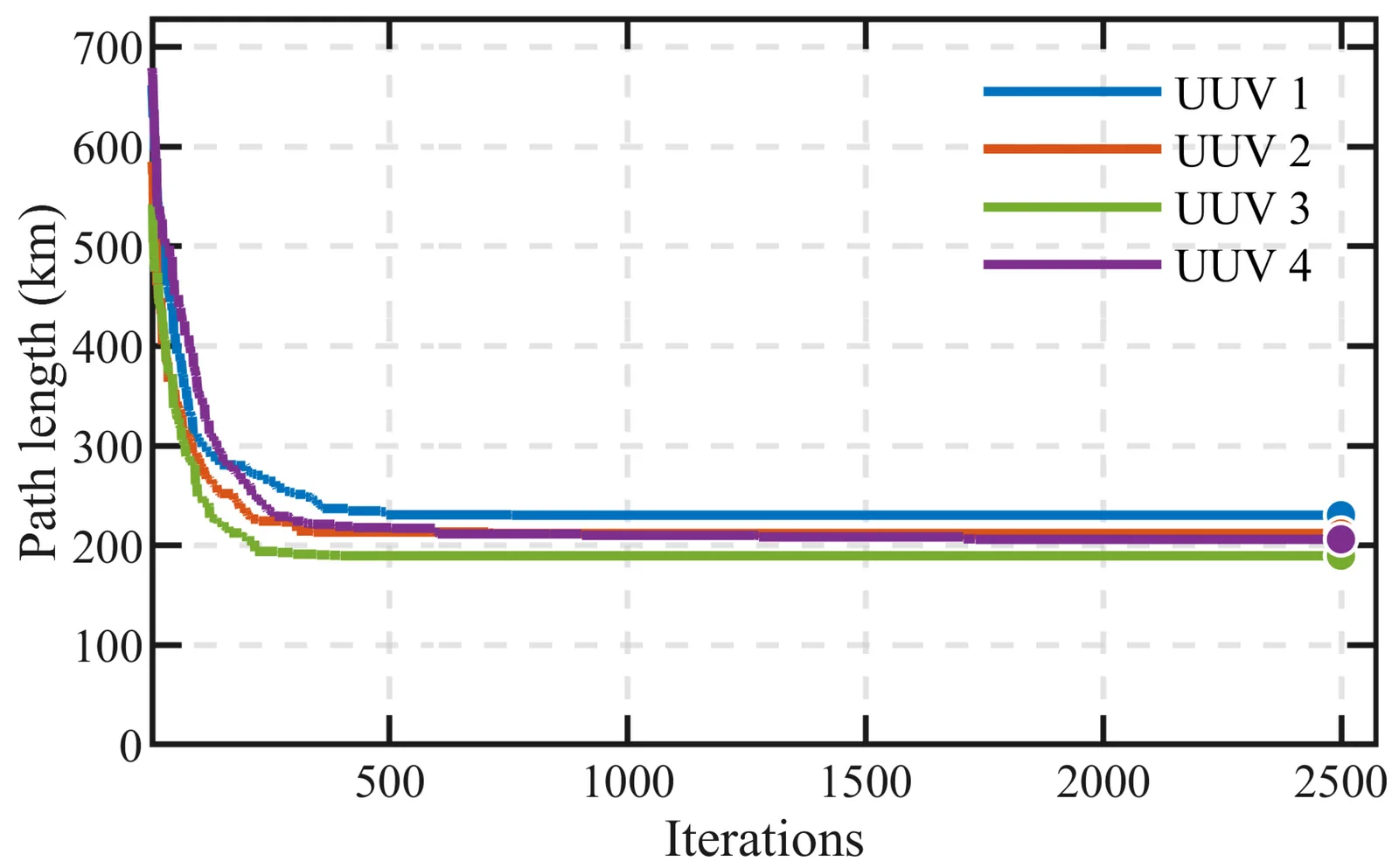
Convergence curve of the genetic algorithm in the rectangular sea area.

To verify the convergence performance of the algorithm in rectangular sea areas, Figure 8 illustrates the variation in the fitness value with iterations in the rectangular sea area ((L, W) = (90, 60) n mile). Within approximately 600 generations, the fitness value drops rapidly from about 680 n mile to about 230 n mile, a reduction of over 66%; between 600 and 1750 generations, the curve exhibits a gradual decreasing trend, slowly optimizing from about 230 n mile to approximately 205 n mile; after 1750 generations, the curve fully stabilizes, indicating that the algorithm has converged to a stable near-optimal solution. The above convergence process verifies that the genetic algorithm can effectively search and stably converge under different aspect ratio conditions in rectangular sea areas.

In the converged solution, the four UUV path lengths are 211.9 n mile, 189.6 n mile, 230.3 n mile, and 206.1 n mile, with a standard deviation of 15.2 n mile, further confirming the algorithm’s load-balancing capability in rectangular sea areas.

### 4.3. Irregular Sea Area

**Figure 9:**
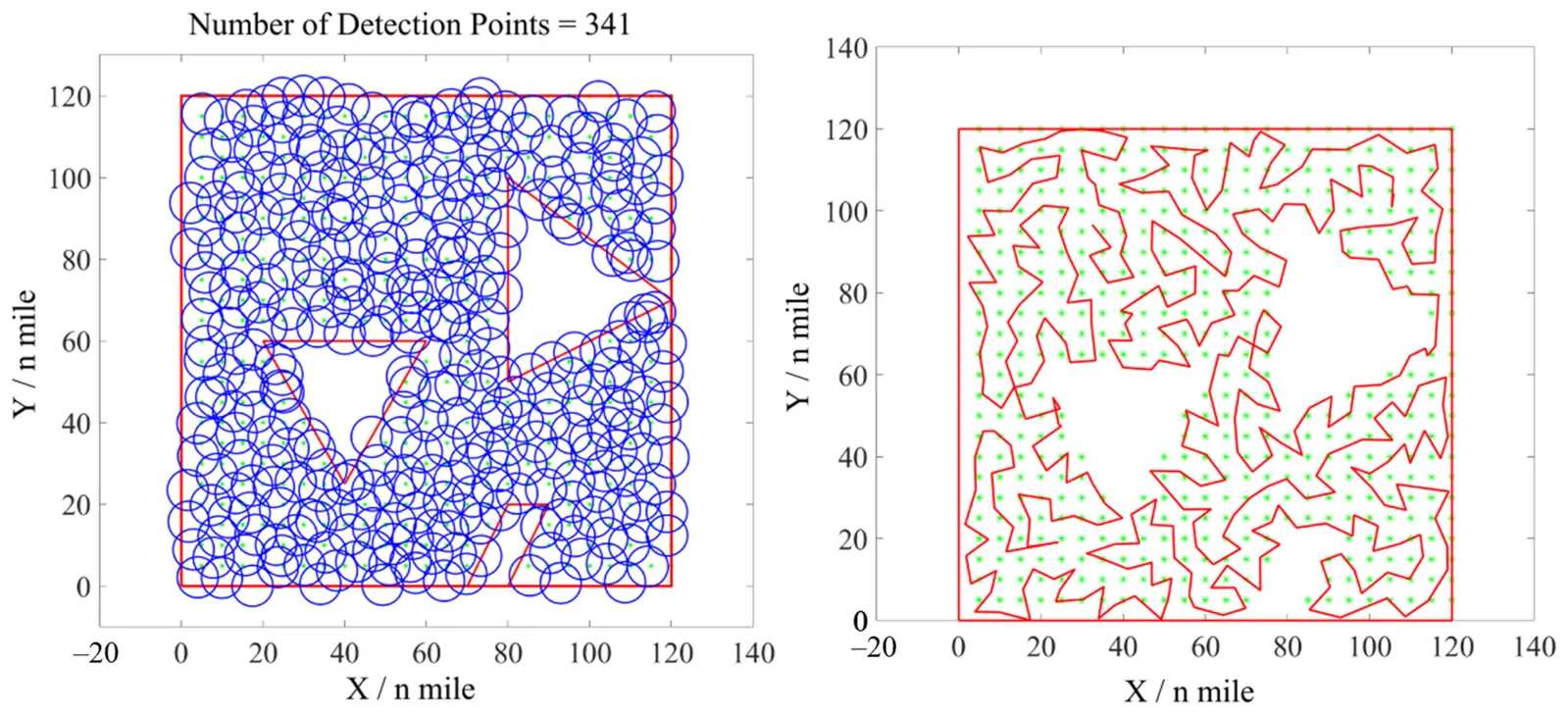
Coverage search scheme and search path in the irregular sea area.

To address the limitations of grid discretization methods or traditional geometric search methods (e.g., parallel, zigzag, and spiral searches) when handling irregularly connected waters, including the difficulty of achieving complete coverage or the issue of path redundancy, this paper carries out simulation experiments in irregular sea area scenarios. The positional parameters of the irregular sea area are defined as follows: within a sea area bounded by [(0, 0), (0, 120), (120, 120), (120, 0), (0, 0)] n mile, there exist three inaccessible areas (islands, reefs, etc.) with coordinates of [(40, 25), (20, 60), (60, 60), (40, 25)] n mile, [(80, 50), (80, 100), (120, 70), (80, 50)] n mile, and [(70, 0), (80, 20), (90, 20), (80, 0), (70, 0)] n mile, respectively, as shown in Figure 9.

In the above irregular sea area scenario, the baseline method plans a coverage search scheme with 376 detection points and a shortest path length of 2196 n mile; the proposed method plans a coverage search scheme with 341 detection points and a shortest path length of 2065.6 n mile. The coverage search scheme and search path are shown in Figure 9.

Table 5 presents a comparison of the baseline method and the proposed method in completing the full-coverage search task. Compared with the baseline method, the number of detection points planned by the proposed method is reduced by 9.3%, and the optimal path length is reduced by 5.9%.

**Figure 10:**
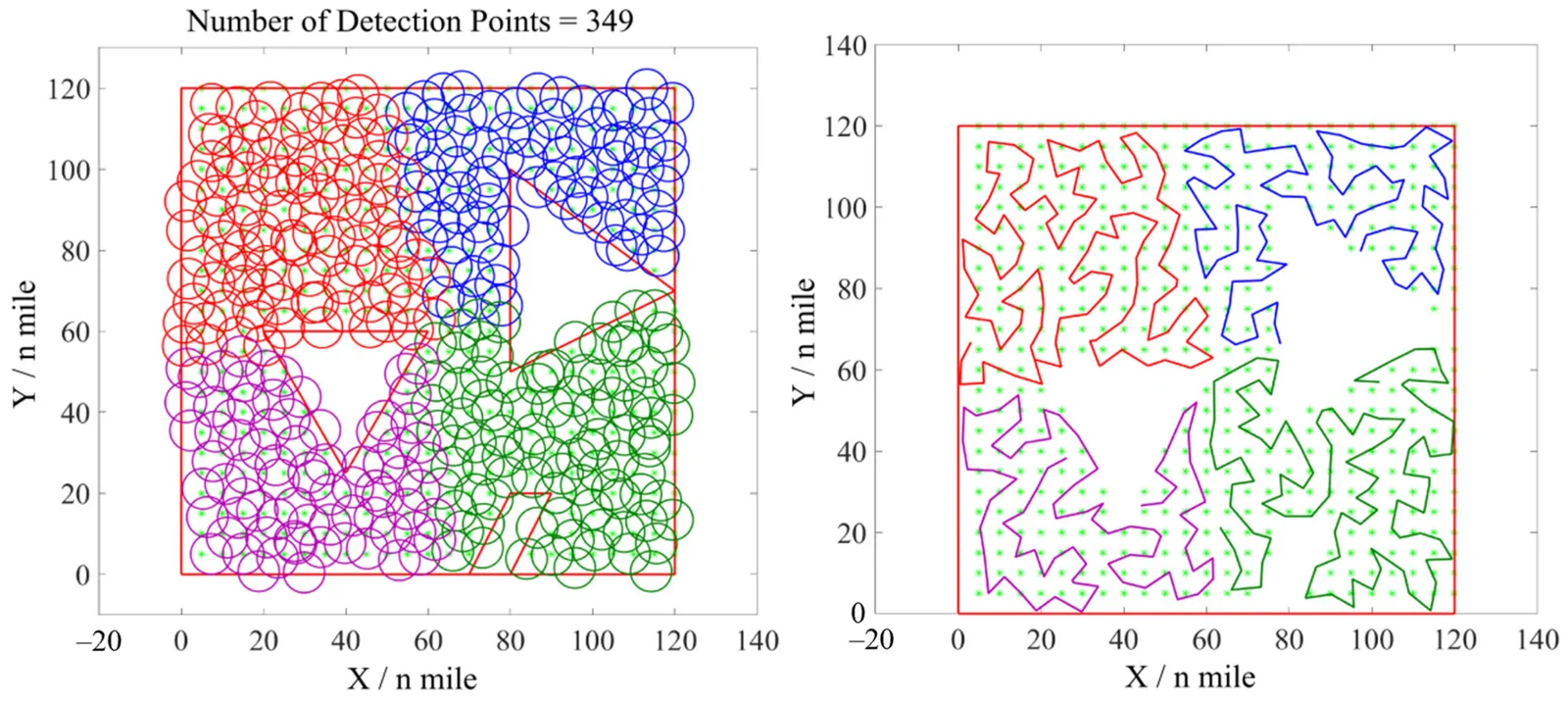
Multi-UUV coverage search scheme and search paths in the irregular sea area.

When four heterogeneous UUVs (two of each type) are used for cooperative search in the irregular sea area, the number of planned detection points is 349 (slightly different from 341 in the single UUV scenario, due to the randomness of Poisson sampling; the median of multiple runs is adopted in this paper). The path lengths of the individual UUVs are 451.5 n mile, 595.1 n mile, 562.6 n mile, and 448.5 n mile, respectively, with a total path length of 2057.7 n mile. The coverage search scheme and the search paths are shown in Figure 10. Compared with the full-coverage task scheme of the baseline method, the total number of detection points is reduced by 7.2%, and the total path length is reduced by 6.3%. When the UUVs travel at a fixed speed, the cooperative operation with four UUVs reduces the completion time for full coverage by 71.2% compared with the single UUV coverage search scheme.

**Figure 11:**
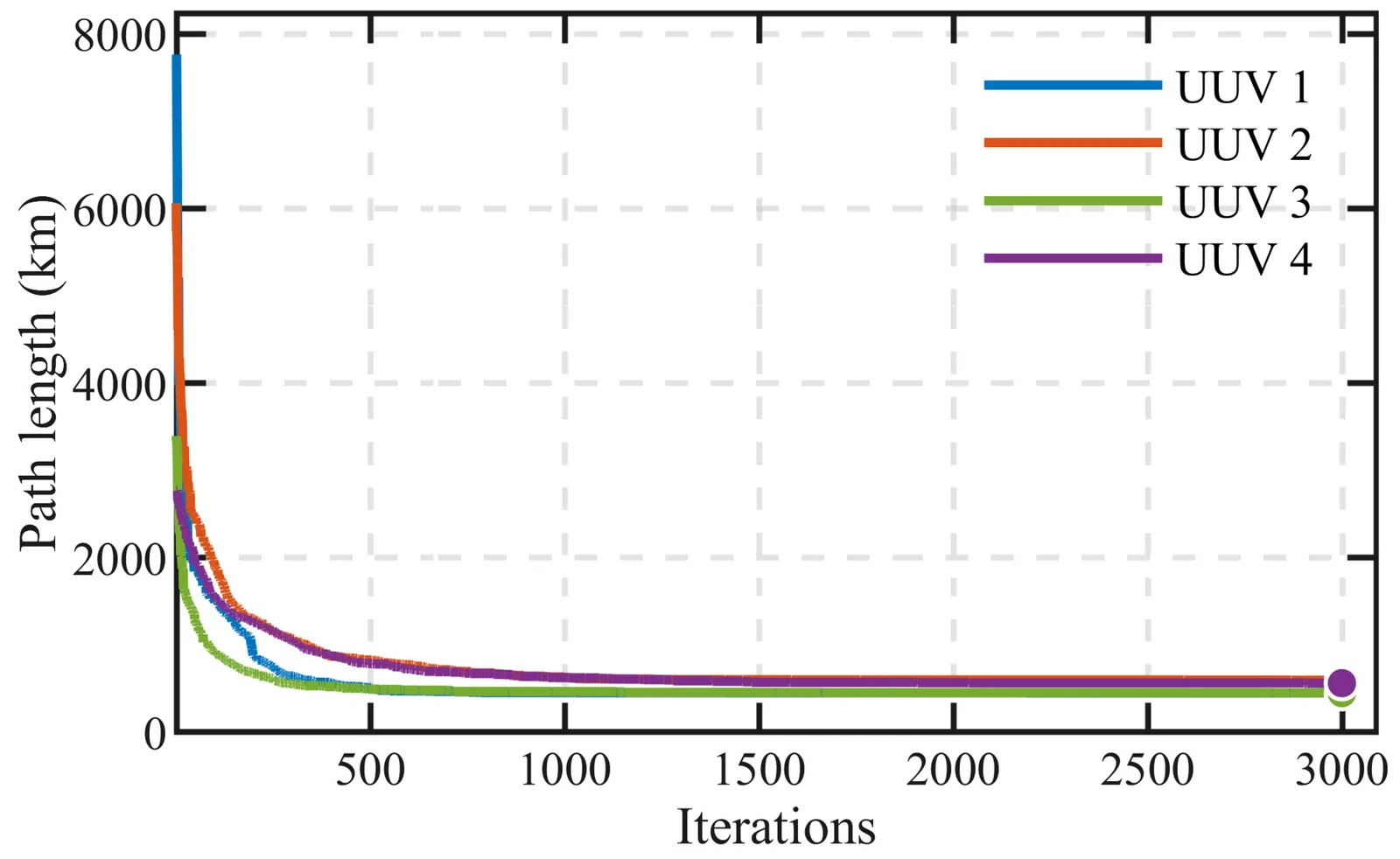
Convergence curve of the genetic algorithm in the irregular sea area.

Regarding the convergence performance in irregular sea area scenarios, Figure 11 presents the variation in the fitness value with iterations. Due to the presence of multiple obstacles in irregular sea areas, the search space is significantly fragmented, substantially increasing the solution difficulty compared with regular sea areas. As can be observed from the convergence curve, within approximately 1500 generations, the fitness value drops rapidly from about 7500 n mile to about 650 n mile, a reduction of up to 90%; thereafter, the curve enters a slow declining phase and gradually converges to approximately 550 n mile. Although the convergence speed is slower than that in regular sea areas, the algorithm is still able to achieve stable convergence within about 2500 generations, indicating that the proposed algorithm maintains good search capability and convergence performance even in complex obstacle-rich environments.

For the irregular scenario, the converged path lengths of the four UUVs are 451.5 n mile, 595.1 n mile, 562.6 n mile, and 448.5 n mile, with a standard deviation of 64.2 n mile. The relatively larger standard deviation, compared with the regular scenarios, reflects the increased difficulty of load balancing caused by obstacle-induced fragmentation of the search space.

### 4.4. Extended Experimental Validation

To further verify the robustness and generalization capability of the proposed algorithm, this paper extends the experimental scenarios from the three typical cases presented in Section 4.1, Section 4.2 and Section 4.3 to 15 distinct instances. The newly added instances are generated by systematically varying the core parameters of each scenario type, with the specific divisions as follows:

(1) Circular sea areas (five instances): The shape is kept fixed while five different radii R = 20, 25, 30, 35, 40 n mile are configured to examine the algorithm’s adaptability to different regional scales.

(2) Rectangular sea areas (five instances): The area is kept approximately constant (≈5400 n mile^2^) while five different aspect ratio configurations (L, W) = (75, 72), (80, 67.5), (90, 60), (100, 54), (108, 50) n mile are set to evaluate the algorithm’s adaptability to different regional shapes.

(3) Irregular sea areas (five instances): The outer boundary is kept fixed while five different obstacle configurations are designed, with the number of obstacles increasing from one to five, to examine the algorithm’s capability in handling environments with increasing complexity of obstacles.

For the above 15 instances, the algorithm is independently run five times for each instance, and the following four key performance metrics are recorded: number of detection points—the total number of detection points deployed by the algorithm to accomplish the full-coverage task, reflecting the task scale and sampling efficiency; longest path length—the maximum single-path length among all UUVs, which directly determines the mission completion time in multi-UUV cooperative missions and serves as the core indicator of the algorithm’s time efficiency; total path length—the sum of all UUV path lengths, reflecting the overall energy consumption; standard deviation of UUV path lengths—this metric is a two-level statistic: first, the standard deviation of all UUV path lengths in a single run (denoted as X_i_) is computed, where a smaller value indicates a more balanced task allocation among UUVs in that run; then, the mean and standard deviation are calculated over X_1_, X_2_, …, X_5_ obtained from five independent runs, and presented in the table as “Mean ± Std.” Here, the mean reflects the central tendency of the load balancing performance over multiple runs, while the standard deviation reflects the stability of the load balancing performance—a smaller standard deviation indicates that the algorithm can stably achieve balanced task allocation under different random conditions. The final results are summarized in Table 6 in the form of “Mean ± Std” to comprehensively reflect the average performance and stability of the algorithm.

From the experimental results presented in Table 6, the following detailed analyses can be derived:

(1) Circular sea area instances (C1–C5): As the sea area radius increases from 20 n mile to 40 n mile, the number of detection points grows from 36.6 to 144.0, which is approximately linearly correlated with the increase in area. This validates that the Poisson-Voronoi hybrid sampling method maintains stable point density control capability across different scales. The longest path length increases from 52.8 n mile to 220.4 n mile, and the mean values of the standard deviation of UUV path lengths remain at a relatively low level of 4.2–13.5, with small standard deviations (i.e., small fluctuations across multiple runs), indicating that the algorithm can stably achieve good load balancing across different scales in circular sea areas.

(2) Rectangular sea area instances (R1–R5): With the area kept approximately constant at about 5400 n mile^2^, the number of detection points remains stable between 150 and 156, with very small fluctuations across instances (standard deviations within 3.7), further validating the algorithm’s stability in point density control. However, as the aspect ratio increases from 1.04 to 2.16, the longest path length gradually rises from 237.5 n mile to 277.8 n mile, and the mean values of the standard deviation of UUV path lengths also increase from 16.0 to 46.9. This trend indicates that the more elongated the regional shape, the more difficult it becomes to achieve balanced UUV task allocation—an inherent structural difficulty of the MTSP in non-compact regions.

(3) Irregular sea area instances (I1–I5): As the number of obstacles increases from 1 to 5, the number of detection points exhibits a non-monotonic trend of first increasing and then decreasing (304.8 → 323.0 → 351.6 → 316.4 → 309.6), indicating that the impact of obstacles on sampling efficiency is not a simple monotonic relationship but is closely related to factors such as the spatial distribution, shape, and degree of fragmentation of the obstacles. The longest path length and total path length also exhibit a similar trend of first rising and then falling, peaking at I3 (with 3 obstacles) at 640.0 n mile and 2252.4 n mile, respectively, before declining in I4 and I5. The mean values of the standard deviation of UUV path lengths are 25.5 in I1, rise to 71.4 in I3, and then decline to 57.1 and 49.4 in I4 and I5. This trend indicates that as the number of obstacles increases from 1 to 3, the connectivity of the sea area is progressively disrupted, increasing the difficulty of task allocation and decreasing load balancing performance. When the number of obstacles further increases to 4–5, the feasible region is more clearly divided into several independent sub-regions, which in turn allows the connectivity-constrained K-means decomposition to more naturally partition the detection points according to spatial connected domains, thereby restoring load balancing to some extent. The above phenomena demonstrate that the key performance metrics of the algorithm are significantly influenced by both the number and spatial distribution characteristics of obstacles, highlighting the engineering significance of properly evaluating algorithm performance in complex marine environments.

In summary, the proposed algorithm stably accomplishes the full-coverage task across all 15 different instances, with the standard deviations of the number of detection points, longest path length, and total path length all maintained within reasonable ranges, indicating that the algorithm maintains good robustness and generalization capability under various conditions including changes in regional scale, shape, and obstacle complexity. The mean values of the standard deviation of UUV path lengths remain at relatively low levels in most instances, with small fluctuations across multiple runs, validating the effectiveness and stability of the dual-chromosome co-evolutionary mechanism in heterogeneous UUV load balancing. It is worth noting that the results from rectangular instance R5 and irregular instance I3 reveal that when the regional shape is extremely elongated or the obstacle layout leads to highly fragmented search areas, the difficulty of load balancing increases significantly—this finding provides a valuable reference direction for future research on multi-UUV task allocation under complex maritime conditions.

## 5. Conclusions

When dealing with full-coverage search tasks, although traditional geometric methods are simple and efficient, they overly rely on regularity assumptions, making it difficult to cope with complex and variable marine environments. Moreover, in practical missions, there exist issues such as differentiated search values in target sea areas and imbalanced path allocation during multi-UUV cooperative search. To address these issues, this paper proposes a heterogeneous UUV full coverage search path planning method that combines a hybrid detection point layout method with a dual-chromosome co-evolutionary genetic algorithm. The main conclusions are as follows:

(1) To overcome the limitations of grid discretization methods or traditional geometric search methods, which often have difficulty in achieving complete coverage, a hybrid detection point layout method integrating Poisson disk sampling and Voronoi diagram gap filling is proposed. On the basis of ensuring a uniform spatial distribution of detection points, this method detects the maximum gap using Voronoi vertices and performs iterative filling, thereby reducing the number of detection points required for the full-coverage task. Simulation results show that in circular, rectangular, and irregular sea areas, the number of detection points planned by this method to achieve full-coverage search of the mission area is reduced by 12.4%, 12.8%, and 9.3%, respectively, compared with the baseline method, significantly reducing the task load.

(2) A co-evolutionary encoding strategy of “task sequence chromosome + split point chromosome” is designed for load balancing of heterogeneous UUVs. Within the same evolutionary individual, the two chromosomes evolve independently via heterogeneous operators but share the same fitness function, enabling simultaneous optimization of path topology and task load distribution. Simulation results demonstrate that the optimal path lengths planned by the proposed method in the three sea area scenarios are reduced by 5.8%, 4.5%, and 5.9%, respectively, compared with the baseline method. When the UUV speed is fixed, the mission times for full-coverage search with four cooperative UUVs are reduced by 73.9%, 72.7%, and 71.2%, respectively, compared with the single UUV case. This indicates that in all three scenarios, the mission time of the coverage search scheme planned by the proposed method is approximately inversely proportional to the number of UUVs, verifying the superiority of the algorithm in terms of balanced task allocation.

(3) Convergence analysis and extended experimental validation further confirm the effectiveness and robustness of the proposed method. The convergence curves of the genetic algorithm in circular, rectangular, and irregular sea areas demonstrate that the fitness value decreases rapidly in the early iterations and gradually stabilizes, with the algorithm reaching stable convergence at approximately 1250, 1750, and 2500 generations, respectively. This convergence behavior substantiates the search effectiveness and solution stability of the algorithm in obtaining high-quality near-optimal solutions. Furthermore, extended experiments on 15 instances—covering five radii in circular areas, five aspect ratios in rectangular areas with approximately constant area, and five obstacle configurations in irregular areas—show that the algorithm consistently maintains the standard deviations of detection point counts, longest path lengths, and total path lengths within reasonable ranges across all instances, with the mean values of UUV path length standard deviations remaining at low levels in most cases, thereby validating the algorithm’s robustness and generalization capability under varying regional scales, shapes, and obstacle complexities. It is worth noting that results from instances with extremely elongated rectangular shapes or highly fragmented irregular regions reveal that load balancing becomes significantly more challenging under such conditions, providing a valuable reference direction for future research on multi-UUV task allocation in complex maritime environments.

## Figures and Tables

**Table 1:** Comparison of our method with representative studies.

Reference	Core Solution Algorithm	Subproblem Composition	Obstacle Avoidance?	Heterogeneous Agents?	Non-Uniform Environment?
Shima et al. [27]	GA	Integrated task and path (no decomposition)	No	No	No
Zhu et al. [28]	Improved SOM + velocity synthesis	Task assignment → Path planning (serial)	Not specified	No	Yes
Xu et al. [29]	CPP + Boustrophedon decomposition	Cell decomposition → Eulerian circuit (serial)	Yes	No	No
Sun et al. [30]	GBNN (neural dynamics)	Direct global coverage (no decomposition)	Yes	No	No
Zhu et al. [31]	SOM with initial heading and current	Task assignment → Path planning (serial)	Not specified	No	Yes
Ye et al. [32]	Modified GA (multi-type-gene encoding)	Task assignment → Dubins path (tightly coupled)	No	Yes	No
Yao et al. [33]	Hierarchical (EM + BN-BMC + AOSC)	Extraction → Allocation → Coverage (serial)	Not specified	No	Yes
Chen et al. [34]	Ant Colony System (ACS)	Region allocation → Order optimization (serial)	No	Yes	No
Hu et al. [35]	Distributed DQN (DCDQN)	Online collaboration (no explicit decomposition)	Yes	No	No
Cheng et al. [36]	DRL + trace pheromone (TP-EDC)	Pheromone-based implicit coordination	Yes	Yes	No
Proposed method	Dual-chromosome co-evolutionary GA	Co-optimization of task allocation and path planning	Yes	Yes	Yes

**Table 2:** Parameter settings for the proposed method.

Category	Parameter	Symbol	Circular	Rectangular	Irregular
Coverage Generation	Detection radius for high-value areas	$r_{L}$	5 km	5 km	5 km
	Detection radius for low-value areas	$r_{H}$	7 km	7 km	7 km
	Poisson sampling grid factor	/	2.3	2.3	2.3
	Voronoi filling max iterations	/	1000	1000	1000
Obstacle Avoidance	Penalty factor	α	1	1	5
Clustering	Number of clusters (UUVS)	m	4	4	4
Genetic Algorithm	Population size	/	80	100	120
	Maximum iterations	/	2500	2500	3000
	Crossover probability	/	0.9	0.9	0.9
	Mutation probability	/	0.2	0.2	0.2
	Elite size	/	5	10	15

**Table 3:** Comparison with the baseline method in a circular sea area.

Search Strategy	Solution Method	Coverage	Number of Detection Points	Path Length /n mile
Baseline method	Numerical solution	Full coverage	97	456.0
Proposed method	Numerical solution	Full coverage	85	429.5

**Table 4:** Comparison with the baseline method in a rectangular sea area.

Search Strategy	Solution Method	Coverage	Number of Detection Points	Path Length /n mile
Baseline method	Numerical solution	Full coverage	180	883.0
Proposed method	Numerical solution	Full coverage	157	843.0

**Table 5:** Comparison with the baseline method in an irregular sea area.

Search Strategy	Solution Method	Coverage	Number of Detection Points	Path Length /n mile
Baseline method	Numerical solution	Full coverage	376	2196.0
Proposed method	Numerical solution	Full coverage	341	2065.6

**Table 6:** Statistical results of algorithm performance under different sea area scenarios.

Scenario Category	Instance ID	Parameter Settings	Number of Detection Points (Mean ± Std)	Longest Path Length/n mile (Mean ± Std)	Total Path Length/n mile (Mean ± Std)	Std of UUV Path Lengths (Mean ± Std)
Circular	C1	R = 20 n mile	36.6 ± 3.6	52.8 ± 3.2	188.3 ± 11.6	4.2 ± 1.9
	C2	R = 25 n mile	61.4 ± 1.5	94.1 ± 6.2	329.5 ± 6.5	8.8 ± 3.5
	C3	R = 30 n mile	88.2 ± 1.1	133.0 ± 3.5	481.4 ± 9.6	11.5 ± 3.2
	C4	R = 35 n mile	114.2 ± 4.1	172.2 ± 8.5	642.0 ± 17.2	9.2 ± 2.5
	C5	R = 40 n mile	144.0 ± 3.7	220.4 ± 9.3	822.5 ± 12.6	13.5 ± 5.3
Rectangular	R1	(75, 72) n mile	153.2 ± 3.7	237.5 ± 7.7	872.0 ± 30.3	16.0 ± 6.7
	R2	(80, 67.5) n mile	150.8 ± 2.9	225.5 ± 7.4	863.4 ± 12.1	6.4 ± 3.9
	R3	(90, 60) n mile	154.0 ± 3.5	234.2 ± 5.3	876.2 ± 16.7	12.8 ± 5.8
	R4	(100, 54) n mile	151.4 ± 3.5	258.0 ± 13.8	871.9 ± 16.4	31.3 ± 11.5
	R5	(108, 50) n mile	156.0 ± 3.1	277.8 ± 13.0	899.8 ± 17.9	46.9 ± 9.2
Irregular	I1	obstacle number = 1	304.8 ± 5.3	480.7 ± 8.8	1785.5 ± 30.6	25.5 ± 2.6
	I2	obstacle number = 2	323.0 ± 5.3	562.9 ± 26.4	1924.2 ± 37.0	54.3 ± 18.7
	I3	obstacle number = 3	351.6 ± 6.2	640.0 ± 25.3	2252.4 ± 103.1	71.4 ± 9.0
	I4	obstacle number = 4	316.4 ± 4.9	591.1 ± 49.5	2039.4 ± 112.0	57.1 ± 21.9
	I5	obstacle number = 5	309.6 ± 4.5	536.7 ± 38.7	1925.3 ± 113.5	49.4 ± 20.3

## Data Availability

The datasets generated and analyzed during the current study are not publicly accessible as they are part of an ongoing research project with confidentiality agreements. For non-commercial research purposes, qualified researchers may request access to the data by contacting the corresponding author (heujifang@163.com) with a detailed research proposal. All requests will be reviewed to ensure compliance with ethical and project-related requirements before data sharing.
